# Intention to have blood-based multi-cancer early detection (MCED) screening: a cross-sectional population-based survey in England

**DOI:** 10.1038/s41416-024-02822-4

**Published:** 2024-08-27

**Authors:** Ninian Schmeising-Barnes, Jo Waller, Laura A. V. Marlow

**Affiliations:** 1 Centre for Cancer Screening, Prevention and Early Diagnosis, Wolfson Institute of Population Health, Queen Mary University of London, London, UK; 2 Cancer Prevention Group, School of Cancer and Pharmaceutical Sciences, King’s College London, London, UK

**Keywords:** Human behaviour, Education

## Abstract

**Background:**

Trials assessing the clinical utility of blood-based multi-cancer early detection (MCED) tests are underway. Understanding public attitudes towards MCED screening is essential if these tests are to be used. We aimed to quantify MCED screening intention and potential barriers and facilitators to uptake.

**Methods:**

Adults aged 50–77 (*n* = 958) completed an online survey. The primary outcome was intention to have MCED screening if offered. Psychological variables including barriers and facilitators were assessed. We used logistic regressions to explore associations between socio-demographics and psychological factors and intention.

**Results:**

93.8% of participants said they would ‘definitely’ or ‘probably’ have MCED screening if offered. Intention was significantly associated with previous screening participation and general cancer attitudes but not with socio-demographic factors. Participants were more likely to be intenders if they had higher health motivation, and perceived greater benefits of blood tests. Participants were less likely to be intenders if they perceived greater disadvantages of blood tests, more practical barriers, were more worried about the outcome and more concerned about a positive result.

**Conclusions and implications:**

MCED screening intention was high. The lack of socio-demographic variation suggests equitable interest in this type of screening; however, future research should consider how intention translates to uptake.

## Background

Earlier stage of cancer diagnosis is generally associated with improved survival [1], as it often increases the chance of successful treatment and outcomes [2]. The NHS Long Term plan set the ambition that 75% of cancers should be diagnosed at stage 1 or 2 by 2028 [3]. Cancer screening aims to identify cancer and pre-cancerous conditions in asymptomatic individuals, but at present only around 6% of cancers are diagnosed through screening in England [4]. Although England currently has three single cancer screening programmes and targeted lung health checks [5, 6], many other cancers have no effective screening test, including aggressive cancers such as pancreatic and stomach cancer. Multi-cancer early detection (MCED) blood tests show potential for detecting cancers before symptoms appear. Blood-based MCED tests look for the presence of circulating tumour DNA or other biomarkers in a standard blood sample and can predict the location of the cancer, in order to direct diagnostic follow-up [7]. A number of MCED tests are in development [8] and trials are currently underway to establish the clinical utility of these tests in asymptomatic individuals, with the hope that MCED tests could be used for population screening in the future [9–11].

The UK National Screening Committee recognises that any new screening programme should be acceptable to the target population and the public [12], since acceptability has implications for engagement, uptake and the overall success of a screening programme [13]. One qualitative study has suggested that the introduction of a population-based MCED screening programme will be appealing due to the simplicity and familiarity of the blood test procedure. However, the potential for positive results and follow-up testing to cause anxiety was an area of concern for participants, and personal beliefs about the benefits of diagnosing cancer early influenced desire for the test [14]. The proportion of people who would want MCED screening, and the barriers and facilitators to this kind of test, have not yet been quantified. It is essential that public attitudes to MCED screening are better understood prior to any future implementation [15].

Many studies investigating the acceptability of screening programmes use intention to participate as a proxy for acceptability [16–19]. Intention has been shown to be a good predictor of future screening uptake, and to be indicative of programme acceptability [20, 21], though there is a gap between intention and behaviour [22]. Quantifying intention can support estimates of cost-effectiveness of a screening programme and plans for resource allocation. There are well-established inequalities in uptake of cancer screening programmes [23], so, research identifying health inequalities should be embedded in the evaluation and introduction of a new screening programme [12]. Understanding the barriers and facilitators to screening participation and whether these might be more/less prevalent among people from lower socio-economic or ethnic minority backgrounds is also important. This can help identify content for information materials to ensure they address questions or concerns that people might face in response to screening invitations and inform proactive interventions to promote equitable uptake.

To our knowledge, no other studies to date have explored intention to participate in population-based MCED blood test screening. Our objectives were: (1) to estimate the proportion of people in a population-representative sample with positive intentions to have an MCED blood test for screening, (2) to quantify the prevalence of barriers and facilitators to having an MCED blood test, (3) to explore socio-demographic predictors of barrier and facilitator endorsement and (4) to explore socio-demographic and psychological predictors of intention to have an MCED blood test.

## Methods

This study is reported following the CROSS reporting guidelines [24] and a copy of the checklist is available in Supplementary material A.

### Design

We ran a cross-sectional online population-based survey of men and women aged 50–77 years in England. The sample was representative of the English population within this age-group in terms of age, sex, ethnicity, social grade and region. Ethical approval for this study was granted by King’s College London Research Ethics committee (LRM-22/23-36139, 06/06/2023). Informed consent was obtained. The study protocol is available at: https://osf.io/zm4v6/.

Data collection was carried out in June 2023 by YouGov. Participants were identified through YouGov’s online research panel. The UK panel includes 2.7 million people recruited via the YouGov website and through social media advertising. Participants receive points for taking part in surveys which can then be exchanged for shopping vouchers.

### Participants

Eligible participants were aged 50–77 years, and currently living in England. This age range matches participants in the ongoing trial of an MCED test (the NHS-Galleri trial; NCT05611632 [11]) and is likely to reflect the population who would be offered MCED blood test screening if it were rolled out at population level. Participants who had taken part in the NHS-Galleri trial and/or had received a cancer diagnosis in the previous three years were not eligible to participate. Potential participants were identified by YouGov from their panel.

Our target sample size was *n* = 1000. This was designed to allow us to estimate intention to have an MCED blood test with good precision (±3%, with 95% confidence, assuming a prevalence of 60–70% for positive intentions).

### Materials and measures

A survey was created by the authors (behavioural scientists) in collaboration with patient and public involvement (PPI) representatives. A detailed description of all survey items is available at: https://osf.io/ka3t7. The survey was only available in English. Participants were shown introductory information about MCED screening followed by additional information about diagnostic work-up following a positive result, including an estimated false positive rate of 0.5% (1 in 200) and a positive predictive value of 50% (1 in 2 with a positive result having cancer); see Box 1. After the introductory information participants answered three knowledge-based attention check questions before completing questions assessing intention, barriers and facilitators. After the additional information about follow-up diagnostic testing, they answered more questions. All questions were mandatory to avoid missing data, except for questions relating to personal details such as health and gender identity, which participants could skip. Participants were excluded from any analyses for which their data were missing.

The full survey is available here: https://osf.io/68wvb. The primary outcome was intention to have MCED screening if offered, measured on a five-point scale with a single item: ‘If you were offered a blood test that looks for a range of cancers would you have it?’ (response options ordered as follows: definitely not; probably not; yes probably; yes definitely and don’t know). Participants who responded probably or definitely not were offered a free-text box to say why. We asked this question twice, at the beginning of the survey after the introductory information (initial intention) and then again after additional information had been provided (considered intention).

The survey included items assessing perceived barriers and facilitators to MCED screening. These items were developed to cover a range of constructs from several theories e.g. the Health Belief Model [25], previous research in the cancer screening context (e.g. [26, 27]) and a qualitative study exploring attitudes to MCED blood tests [14]. It also contained items assessing cancer-related attitudes: attitudes to screening [28], cancer fatalism [29], cancer risk perceptions [30, 31] and attitudes to overdiagnosis [28]. Detail about the source of each item is in the protocol (https://osf.io/ka3t7).

We adopted YouGov’s standard socio-demographic questions for age, ethnicity, social grade (based on occupation of the highest household earner), and region, with additional items from the UK census included to assess sex, marital status, education and self-reported disability. We also assessed previous screening behaviour (items adapted from the Cancer Awareness Measure [26]), and global self-rated health [32]. The survey items were a combination of validated and unvalidated measures and were assessed for face validity via PPI feedback.

Box 1 Information presented to participants about MCED tests within the survey ***Initial information:***

This survey is about your thoughts on a new blood test that looks for a range of cancers. This type of test looks for cancer markers in a blood sample. This is called a Multi Cancer Early Detection (MCED) blood test.

This test would be a normal blood test. A health professional would take a small tube of blood and send this to a lab for testing.

This would be a screening test for healthy people without symptoms. The test would be used alongside existing cancer screening.

If cancer markers are found, you would need to have follow-up tests at a local hospital to see if you have cancer. About half of the people who are sent for further tests are expected to have a cancer found.

***Further information:***

If a cancer marker is found in your blood, you would need to have follow-up tests (e.g. scans) at a local hospital to see if you have cancer.

Imagine that 200 people have this test: 2 out of 200 people will have a cancer marker found in their blood. 1 of these would have cancer diagnosed after further tests and 1 would not.

(This is an estimate, we do not know exactly how these tests will perform yet).

### Procedure

Participants were sent an email including a unique re-direct link taking them to the survey. This allowed YouGov to link responses to socio-demographic data and avoided multiple submissions. Participants who clicked the link were shown a participant information page and asked to indicate consent to take part.

When participants completed the survey, they were thanked for their participation and offered sources of support. Participants received 150 YouGov points (approximately £1.50) upon completion of the survey.

### Analysis

All datasets were anonymous. The data were analysed using the Complex Samples functionality in IBM SPSS Statistics 29 and followed a pre-written analysis plan (available here: https://osf.io/zm4v6/). Deviations from this plan were recorded in a separate document (https://osf.io/zm4v6/). Weights were provided by YouGov to account for outstanding variation between the recruited sample and the wider population with respect to age, sex, social grade, region and ethnicity so that the sample was representative of English adults aged 50–77 years [33]. All analyses were weighted so that the sample was population representative. Participants were excluded prior to analyses if they completed the survey too quickly (in less than 5 min), too slowly (in more than 3 standard deviations above the mean time taken) or if they answered all three attention check questions incorrectly.

Frequencies and proportions (with 95% confidence intervals) of individuals giving each response option for the two intention items (initial and considered) were reported. For further analysis exploring intention, a single binary variable was created using considered intention and classifying participants as ‘intenders’ or ‘non-intenders/don’t knows’. We used logistic regression to assess whether intention (using the binary outcome) was associated with socio-demographic characteristics.

The frequency and proportion of individuals responding ‘agree’/‘strongly agree’ or ‘quite a bit’/‘a great deal’ to each barrier/facilitator item is reported (full breakdown of responses available in Supplementary Tables 1 and 2). Following Exploratory Factor Analysis (EFA, see supplementary material), the 21 items assessing barriers and facilitators were reduced to five scales assessing: Health Motivation (4 items; Cronbach’s alpha = 0.85); Benefits of blood tests (4 items; Cronbach’s alpha = 0.87); Disadvantages of blood tests (4 items, Cronbach’s Alpha = 0.80); Practical barriers (3 items, Cronbach’s alpha = 0.71) and Fear of outcome (3 items, Cronbach’s alpha = 0.82). The six items assessing concern about a positive result formed an additional scale (Cronbach’s alpha = 0.90). Scales were created by summing the items and adjusting for a minimum score of zero. Three items did not fit in the scales and were analysed individually. We used fully adjusted ANCOVAs to assess whether age, sex, social grade, ethnicity or employment status were independently associated with barriers/facilitators scores. For the three single item barriers, we ran logistic regressions to examine demographic associations. We used a Bonferroni correction to adjust for multiple comparisons (45 in total), resulting in a p-value of <0.001 being considered statistically significant.

We used logistic regression to assess whether intention (using the binary outcome) was associated with any of the psychological variables including: perceived risk of cancer, cancer worry, fatalism, attitudes to screening/overdiagnosis and the six scales or three individual items assessing barriers/facilitators. We used content analysis to analyse the free text responses recorded by participants who responded ‘probably not’ or ‘definitely not’ to the intention item. Two authors worked together to develop a coding frame and independently coded each response. Discrepancies were discussed and resolved.

### Public and patient involvement (PPI)

A PPI panel consisting of five participants was involved in the development of the participant information page, consent form, and survey. The panel included two women and three men aged 50–70 years and included people with and without personal cancer experience. Development of the research questions was carried out before recruitment of the PPI panel. Our PPI panel had substantial influence on the information that was presented and the need to split this into different sections. Discussion also included in-depth debate about the best wording for items and their response options. The discussions provided an indication of the face validity of newly created items, and ensured they were presented in a logical and user-friendly way.

## Results

### Sample

Of the 1250 people who clicked the survey link, 1052 were eligible and consented (representing an 84.2% completion rate based on survey completes/survey started). The survey was fully completed by 1002 participants but 44 were excluded prior to analysis (*n* = 10 for answering all three attention check items incorrectly; *n* = 34 for completing the survey too quickly/slowly). Data from 958 participants was included in the analysis. Sample characteristics are presented in Table 1.

**Table 1:** Sample characteristics.

	Unweighted (*n* = 958)		Weighted (*n* = 957)	
	*n*	%	*n*	%
Sex (*n*; %)				
Female	500	52.2	496	51.8
Male	458	47.8	462	48.2
Age (*n*; %)				
50–59	409	42.7	413	43.2
60–69	326	34	322	33.6
70–77	223	23.3	222	23.2
Social Grade (*n*; %)				
A/B (highest)	237	24.7	214	22.3
C1	274	28.6	281	29.4
C2	200	20.9	219	22.8
D/E (lowest)	247	25.8	244	25.5
Ethnicity (*n*; %)				
White	855	89.2	852	89
Black, Black British, Caribbean or African	37	3.9	38	4
Asian, Asian British	36	3.8	36	3.8
Mixed or Multiple	23	2.4	24	2.5
Other ethnic group	7	0.7	7	0.7
Highest level of education^a^ (*n*; %)				
No formal qualifications	56	5.8	57	5.9
Level 2	217	22.7	220	23
Apprenticeships	29	3	29	3
Level 3	225	23.5	227	23.7
Level 4	277	28.9	270	28.2
Other	131	13.7	130	13.6
Employment status (*n*; %)				
Working	450	47	456	47.6
Not working	485	50.6	479	50
Marital status (*n*; %)				
Married/Civil partnership/Living as married	623	65	622	65
In a relationship (not living together)	30	3.1	31	3.2
Single (never married)	121	12.6	123	12.8
Separated/divorced/widowed	177	18.5	175	18.3
Ever participated in bowel screening (*n*; %)^b^	569^b^	59.4	564	58.9
Ever attended breast screening (*n*; %)^c^	445^c^	89.4	440	89.2
Ever attended cervical screening (*n*; %)^d^	477^d^	95.8	473	95.8
Self-rated health (*n*; %)				
Poor/fair	409	42.7	409	42.7
Good/very good/excellent	551	56.5	541	56.4
Self reported disability (*n*; %)	339	35.4	338	35.3

^a^LEVEL 2 included City and Guilds certificate, CSE grade 1, GCE O level, GCSE school certificate or Scottish Ordinary/Lower Certificate; APPRENTICESHIPS included recognised trade apprenticeships or clerical and commercial qualifications; LEVEL 3 included City and Guilds advanced, ONC, University diploma, GCE A level or Higher Certificate or Scottish Higher Certificate; LEVEL 4 included Nursing qualifications, teaching qualification (not degree), University or CNAA first degree, University or CNAA higher degree.

^b^denominator included all participants who responded to the question, *n* = 948 (*n* = 3 who responded ‘don’t know’ and *n* = 7 who missing were excluded)

^c^Question asked to women only, denominator included all women who answered the question, *n* = 498

^d^Question asked to women only, denominator included all women who answered the question, *n* = 498 (*n* = 1 who responded ‘don’t know’ was excluded)

### Intention to have MCED screening

Initially, most participants said they would ‘probably’ (*N* = 299; 31.3%) or ‘definitely’ (*n* = 595; 62.2%) have MCED screening (initial intention) with a slight increase in intention strength after reading additional information, (*n* = 257; 26.9% and *n* = 641; 66.9% respectively). All 36 people who said they would probably or definitely not have the test after the initial intention question recorded a free-text response. Participants gave a range of different reasons for their response. The most frequent were: ‘test causing anxiety’ (*n* = 12), ‘not wanting to know about cancer’ (*n* = 7), ‘low perceived need for the test’ (*n* = 7) and ‘wanting to focus on living life’ (*n* = 5) (see Supplementary Table 3 for all responses).

Using the binary outcome (intenders versus non-intenders/don’t know), we did not find significant associations between intention to have MCED screening and any of the socio-demographic characteristics we assessed (age, sex, social grade, ethnicity, education, employment and marital status) or with self-rated health (see Table 2). Of the 860 participants who were eligible for any of the three screening programmes, those who reported never attending any screening or only attending some screening were less likely to be intenders than those who had attended all the screening they were eligible for (*n* = 5/7; 71.2% who had never attended and *n* = 172/203; 84.7% who had attended some compared with *n* = 629/650; 96.6% who had attended all). Those who reported ‘a bad experience’ of screening in the past were less likely to be intenders (83.0%) than those who had not (94.4%)

**Table 2:** Unadjusted logistic regression analyses exploring the association between socio-demographics, health behaviours, perceived risk of cancer, cancer worry and fatalism and intention to have an MCED test (weighted).

	Intenders^a^	Unadjusted	Wald F (df), *p* value
	*n* (%)	OR (95% CI)	
All	**898 (93.8%)**		
Age			
50–59	386 (93.4)	1	1.278 (2956), *p* = 0.278
60–69	298 (92.8)	0.91 (0.51–1.63)	
70+	213 (96.1)	1.72 (0.79–3.73)	
Sex			
Male	439 (95.0)	1	2.03 (1957), *p* = 0.154
Female	460 (92.7)	0.68 (0.39–1.16)	
Social Grade			
A-B	205 (95.8)	1	2.57 (3955) *p* = 0.053
C1	268 (95.3)	0.89 (0.38–2.07)	
C2	206 (94.0)	0.70 (0.29–1.65)	
D-E	220 (90.2)	0.41 (0.190–0.873)	
Ethnicity			
White	803 (94.2)	1	0.17 (3948) *p* = 0.919
Black	23 (96.2)	1.54 (0.20–11.71)	
Asian	34 (94.5)	1.06 (0.24–4.49)	
Mixed	35 (92.1)	0.71 (0.21–2.40)	
Highest level of education			
No formal qualifications	52 (91.0)	0.60 (0.22–1.78)	1.11 (5930) *p* = 0.353
Level 2	207 (94.0)	0.97 (0.46–2.08)	
Apprenticeships	28 (96.7)	1.81 (0.23–14.24)	
Level 3	217 (95.6)	1.33 (0.59–3.00)	
Level 4	254 (94.2)	1	
Other	117 (89.9)	0.55 (0.26–1.19)	
Employment status			
Working	428 (94.0)	1	0.01 (1, 934) *p* = 0.925
Not working	451 (94.2)	1.03 (0.59–1.77)	
Marital status			
Married/Civil partnership/Living as married	585 (94.1)	1	0.04 (3,948), *p* = 0.990
In a relationship (not living together)	29 (93.2)	0.87 (0.20–3.79)	
Single (never married)	114 (93.4)	0.89 (0.40–1.97)	
Separated/divorced/widowed	164 (93.8)	0.96 (0.48–1.92)	
Screening experience			
Attended all eligible	629 (96.6)	1	**17.60 (2861),** ***p*** < **0.001**
Attended some eligible	172 (84.7)	0.19 (0.11–0.34)	
Never attended	5 (71.2)	0.09 (0.02–0.47)	
Past bad experience of cancer screening			
Yes	39 (83.0)	0.29 (0.13–0.66)	**8.86 (1957)** ***p*** = **0.003**
No	859 (94.4)	1	
Self-rated health			
Poor/fair	383 (93.7)	0.985 (0.58–1.68)	0.003 (1949) 0.957
Good/very good/excellent	507 (93.8)	1	
Perceived risk of cancer			
Above average	192 (93.4)	0.22 (0.11–0.46)	**9.70 (2947)** ***p*** < 0.001
Same as average	627 (95.4)	1	
Below average	70 (82.2)	0.68 (0.35–1.32)	
Cancer worry frequency			
Never/Rarely/Sometimes	815 (94.4)	1	**5.12 (1957)** ***p*** = **0.024**
Often/Very often	83 (88.4)	0.45 (0.22–0.90)	
Cancer Fatalism			
*Predetermination*			
Yes	167 (86.4)	0.29 (0.17–0.50)	**20.24 (1957)** ***p*** < **0.001**
No	732 (95.7)	1	
*Incurability*			
Yes	7 (76.5)	0.21 (0.04–1.03)	3.72 (1957) *p* = 0.054
No	891 (94.0)	1	

^a^responded ‘yes, definitely’ or ‘yes, probably’ to the considered intention item (after all information had been presented). Bold indicates significance at <0.05

### Barriers and facilitators to MCED screening

The percentage of participants who endorsed each barrier and facilitator to MCED screening is presented in Table 3. The most frequently endorsed barriers were: being frightened about what the test might find (45.0%), needing to know more about how the test works (39.6%) and the potential for MCED screening to cause worry about cancer (32.0%). The least frequently endorsed barrier to MCED screening was being ‘too busy’ (1.2%). The most frequently endorsed facilitators to MCED screening related to blood tests being safe (92.7%), quick (92.9%) and familiar (86.7%).

**Table 3:** Summary descriptives for each barrier/facilitator item and composite scale (weighted n = 957).

	% (95% CI) for item	Mean (SD) for scale
*Health motivation* (scale, range 0–16)^a^		12.62 (2.69)
This test would make me feel I was doing something positive about my health	86.5 (84.1–88.5)	
I would want this test even if I felt healthy	82.5 (79.9–84.8)	
This test would give me reassurance	79.1 (76.4–81.6)	
I would not need a test if I did not have any symptoms	3.0 (2.1–4.3)	
*Benefits of blood tests* (scale, range 0–16)^a^		12.99 (2.73)
Blood tests are familiar to me	86.7 (84.4–88.8)	
I am used to having blood tests	76.6 (73.7–79.1)	
Blood tests are quick	92.9 (91.1–94.4)	
Blood tests are safe	92.7 (90.9–94.2)	
*Disadvantages of blood tests* (scale, range 0–16)^a^		4.25 (3.43)
I am afraid of needles	12.5 (10.6–14.8)	
I find blood tests uncomfortable	19.7 (17.3–22.4)	
Blood tests are painful	8.2 (6.6–10.1)	
I am afraid of the sight of my own blood	7.4 (5.9–9.3)	
*Fear of outcome* (scale, range 0–12)^a^		5.44 (3.09)
I would be frightened of what the test might find	45.0 (41.9–48.2)	
This test would make me worry about having cancer	32.0 (29.1–35.1)	
I would be afraid of having treatment if cancer was found	29.2 (26.4–32.2)	
*Practical barriers* (scale, range 0–12)^a^		2.15 (1.99)
I would find it difficult to get an appointment at a time that suits me	10.3 (8.5–12.4)	
I would find it difficult to travel to an appointment at my GP surgery	3.2 (2.3–4.6)	
I would be too busy to have a blood test	1.2 (0.6–2.1)	
*Concern about a ‘positive’ result* (scale, range 0–24)^b^		4.74 (4.41)
Feeling worried about having a cancer marker found in my blood	24.9 (22.3–27.8)	
Needing to have follow-up tests if a cancer marker was found	19.6 (17.2–22.3)	
Being anxious if I needed follow-up tests	25.3 (22.7–28.2)	
Needing to have a scan at a hospital	12.8 (10.9–15.1)	
Needing to have an endoscopy (where a small camera is put inside your body)	29.2 (26.4–32.2)	
Needing to have follow-up tests and then no cancer being found	9.9 (8.2–12.0)	
*Individual items*^a^		
I would need to know more about how the test works	39.6 (36.5–42.8)	
I would not trust the blood test results	2.8 (1.9–4.0)	
I would have more important things to worry about than this test	7.4 (5.9–9.3)	

^a^% of participants who responded agree or strongly agree (items assessed after introductory information);

^b^% of participants who felt this would put them off MCED screening ‘quite a bit’ or ‘a great deal’ (items assessed after additional information).

Note: The number and percentage of participants indicating each response are available as in Supplementary Tables 1 and 2.

We looked at whether socio-demographic characteristics (age, sex, social grade, ethnicity and employment status) were associated with any of the six composite scales (Supplementary Tables 6 and 7). Scores for the ‘practical barriers’ were significantly higher in individuals from lower social grades (C1, C2 and D/E) compared to those from the highest social grade (A/B; *p* < 0.001). Older age groups had lower ‘fear of outcome’ scores compared with the youngest group (*p* < 0.001). Participants from ethnic minority backgrounds were also more likely to say that they would need more information than participants from white ethnic backgrounds (*p* < 0.001).

### Psychological predictors of intention

General beliefs about cancer risk, fatalism and general attitudes to cancer screening were significantly associated with intention to have MCED screening (see Tables 2 and 4). Participants were less likely to be intenders if they felt their risk of cancer was ‘above average’ or ‘below average’ (82.2%) versus ‘same as average’ (95.4%), and if they worried about cancer ‘often’ or ‘very often’ (88.4%) versus ‘never’/‘rarely’/‘sometimes’ (94.4%). Participants who believed cancer is predetermined were also less likely to be intenders (86.4%) than those who did not hold this fatalistic belief (95.7%).

**Table 4:** Unadjusted logistic regression analyses exploring the association between attitudes to screening/overdiagnosis and intention to have an MCED test (weighted).

	Intenders (*n* = 898) *n* (row %)	Unadjusted OR (95% CI)	Chi-squared (df), *p* value
Cancer screening tests for healthy people are almost always a good idea			
Yes	791 (97.2)	1	**74.23 (1957)** ***p*** < **0.001**
No	107 (74.7)	0.08 (0.05–0.15)	
Finding cancer early almost always means that treatment saves lives			
Yes	721 (96.7)	1	**39.092 (1957)** ***p*** < **0.001**
No	177 (83.7)	0.18 (0.10–0.30)	
Finding cancer early almost always means that a person can have less treatment			
Yes	578 (96.7)	1	**19.842 (1957)** ***p*** < **0.001**
No	320 (89.1)	0.28 (0.16–0.49)	
If there was a kind of cancer for which nothing could be done, I would want to be tested to see if I had it			
Yes	606 (99.5)	1	**37.47 (1957)** ***p*** < **0.001**
No	292 (83.9)	0.03 (0.01–0.08)	
If I had early-stage cancer, I would want to have the recommended treatment			
Yes	851 (96.4)	1	**81.78 (1957)** ***p*** < **0.001**
No	47 (63.3)	0.07 (0.04–0.12)	
I would want to be tested to see if I had cancer even if it was slow-growing and would not cause me harm in my lifetime			
Yes	751 (98.3)	1	**79.71 (1957)** ***p*** < **0.001**
No	147 (76.0)	0.05 (0.03–0.10)	

Bold indicates significance at <0.05.

Participants were more likely to be intenders if they believed: cancer screening is always a good idea, that finding cancer early means that treatment saves lives, or that finding cancer means a person has less treatment (*p* < 0.001). Views on overdiagnosis were also associated with intention, with participants more likely to be intenders if: they said they would want to be tested for cancer, even if nothing could be done; they said they would still have screening, even if it was for a slow-growing cancer that would not cause them harm in their lifetime; they said they would have the recommended treatment for early-stage cancer (*p* < 0.001).

Using the six barrier and facilitator scales we looked at the association between barriers and facilitators and intention. In unadjusted analyses, all scales were significantly associated with intention to have MCED screening (*p* < 0.001) (Table 5). Higher ‘Health motivation’ and ‘Benefits of blood tests’ scores were associated with increased odds of being an intender. Conversely, higher scores on ‘Disadvantages of blood tests’, ‘Fear of outcome’, ‘Practical barriers’ and ‘Concerns about a ‘positive’ result’ were associated with decreased odds of being an intender. Participants who said that they would need more information (*F*(1957) = 16.15 *p* < 0.001), that they wouldn’t trust the results (F(1957) = 30.79 *p* < 0.001), or that they had more important things to worry about (F(1957) = 24.83 *p* < 0.001) were less likely to be intenders. After adjusting for all the barriers and facilitators (scales and items), three scales remained significant predictors of intention to have MCED screening: health motivation (F(1957) = 64.37 *p* < 0.001), practical barriers (F(1957) = 6.19 *p* = 0.013) and concerns after results (F(1957) = 16.66 *p* < 0.001) (Supplementary Tables 4 and 5).

**Table 5:** Unadjusted logistic regression analyses exploring the association between barriers and facilitators and intention to have MCED screening (weighted).

	Intenders (*n* = 898)	Non-intenders (*n* = 59)	Unadjusted OR (95% CI)	Chi-squared (df), *p* value
	Mean (SD)	Mean (SD)		
Health Motivation (scale, range 0-16)	12.98 (2.27)	7.20 (2.74)	2.29 (1.96-2.68)	**109.75 (1957)** ***p*** < 0.001
Benefits of blood tests (scale, range 0-16)	13.15 (2.64)	10.54 (2.93)	1.34 (1.23-1.47)	**41.23 (1957)** ***p* < 0.001**
Disadvantages of blood tests (scale, range 0-16)	4.13 (3.35)	6.21 (4.02)	0.85 (0.79-0.92)	**17.73 (1957)** ***p* < 0.001**
Fear of outcome (scale, range 0-12)	5.23 (3.00)	8.56 (2.82)	0.65 (0.56-0.74)	**37.35 (1957)** ***p* < 0.001**
Practical barriers (scale, range 0-12)	1.98 (1.84)	4.61 (2.51)	0.58 (0.51-0.66)	**69.22 (1957)** ***p* < 0.001**
Concern about a positive result (scale, range 0–24)	4.27 (3.98)	11.90 (4.40)	0.72 (0.68-0.76)	**111.97 (1957)** ***p* < 0.001**
	*n* (% agree)	*n* (% agree)		
Need more information	340 (37.9)	38 (65.6)	0.32 (0.18-0.56)	**16.15 (1957)** ***p* < 0.001**
Would not trust results	17 (1.9)	10 (3.2)	0.09 (0.04-0.21)	**30.79 (1957)** ***p* < 0.001**
More important things to worry about	56 (6.2)	15 (25.6)	0.19 (0.10-0.37)	**24.83 (1957)** ***p* < 0.001**

Bold indicates significance at <0.05.

## Discussion

In this population-based survey in England, most people said they would have MCED screening and intention remained high after receiving additional information about diagnostic work-up following a positive result and the potential for false-positive results. Intention to have MCED screening was not associated with socio-demographic characteristics but did vary according to previous screening behaviours and attitudes to cancer and screening in general. General health motivation, anticipated practical barriers and concerns about what would happen following a positive result, were the strongest predictors of intention to have MCED screening. The need for additional information, fear of the outcome and concern about what would happen after results were endorsed by a sizeable minority (i.e. over a third of participants).

This is the first study to quantify intention to have MCED screening in a sample representative of the English population following provision of brief information. The findings add to evidence from a 2021 report investigating public priorities in cancer research which reported that 70% of participants would want a single blood test for multiple cancers, but they were not given any further information about blood tests for cancer screening [34]. Since intention is a strong predictor of uptake [20], the high intention demonstrated in our study, following brief information, suggests MCED screening uptake could be high if rolled out across England. Nevertheless, it is important to consider that intention does not always translate into action in cancer screening contexts [22]. For example, in colorectal cancer screening 80% of people who said they would probably or definitely have screening subsequently attended flexi-sigmoidoscopy [21]. Future research is needed to assess how intention translates to action in the context of MCED screening and factors influencing this. The finding that intention did not vary by socio-economic and demographic characteristics suggests motivation to have MCED screening may be equally high across socio-demographic groups. Nevertheless, as outlined in widely used behaviour change frameworks (e.g. COM-B [35]), capability (whether someone has the knowledge, skills and abilities to engage in particular behaviour) and opportunity (external factors influencing whether a behaviour can be executed) are also important determinants of health behaviour.

Previous screening behaviour significantly predicted intention to have MCED screening, consistent with previous research in other screening contexts [19, 36]. MCED screening does hold potential to engage people whose previous non-attendance/participation is driven by aversion to more invasive procedures, and intention to have an MCED test was still high in those who had never or inconsistently attended (71% and 85% respectively). This suggests uptake may be high even in those who do not participate in other screening programmes. However, individuals who had never or inconsistently attended screening were still less likely to intend to have MCED screening. Non-intenders were also less likely to support screening in general and scored significantly lower on the ‘health motivation’ scale. This suggests that for some, cancer screening is not in line with their values, and if this is the case it makes sense that previous behaviour would continue to predict intention to some degree. This finding has also been observed in qualitative work [14]. An individual’s informed choice not to have MCED screening should be respected. Nevertheless, efforts should be made to ensure that never or infrequent screening participation is a choice, and not the result of specific, modifiable barriers to screening such as lower knowledge or fatalistic attitudes that could warrant intervention.

The benefits of blood tests as a procedure were highly endorsed with few perceiving disadvantages. This supports qualitative findings suggesting that the primary test procedure will be appealing to most people [14]. Individuals who were non-intenders were more likely to be concerned about a positive result, the need for follow-up testing and the potential for this to cause anxiety. If MCED screening is rolled out in the future, it will be important to manage these concerns and detailed information materials should be developed to give reassurance regarding follow-up tests, since it seems these could put individuals off MCED screening at the point of invitation. Interestingly, providing participants with estimated detail about the positive predictive value of an MCED test (i.e. ‘2 out of 200 people will have a cancer marker found in their blood. 1 of these would have cancer diagnosed after further tests and 1 would not’) did not decrease intention, in fact it was stronger after this information was provided. Research exploring attitudes to mammography suggests that people are extremely tolerant of false-positive results [37]; however, further work is needed to explore public tolerance of false-positive results in MCED testing, for finding cancer overall and for different types of cancer. We did not present information about false negative results (of which there is much lower public tolerance), since it is not possible to make an estimate about this at the moment.

Another frequently cited barrier was the need for additional information. This finding is not surprising since MCED screening is still relatively unheard of, and participants received limited information within the survey. If implemented, participants will likely be given comprehensive informational resources at the time of invitation to support informed choice, as seen in other national screening programmes [38–40]. Materials outlining the benefits and harms of screening, and explaining procedural elements, are highly valued by potential participants, as has been reported elsewhere [41–43]. At present there is limited information available about MCED tests [44]. Care and thorough testing should be undertaken to ensure that sufficiently detailed and accessible materials answering relevant questions are available.

Whilst motivation did not vary by demographic characteristics, some of the barriers and enablers were more prominent in particular sub-groups. Average scores on the ‘fear of the outcome’ scale were higher for younger than older participants. This is in line with some evidence suggesting that cancer worry decreases with age [45], although this relationship has been refuted in other studies [46]. This suggests that interventions designed to reassure people about the treatability of cancer and emphasise good survival outcomes could be particularly important for younger participants. Practical barrier scores were higher in those from lower occupational social grades. Other studies have found that practical barriers to screening such as appointment times, lacking spare time [47] and transport issues [48] are more prevalent in individuals from more deprived backgrounds. If MCED screening is implemented, flexible appointments should be offered in accessible locations and booking processes should be simplified to reduce inequity.

### Strengths and limitations

The use of a sample that was population-representative with respect to age, sex, ethnicity, social grade and region should allow the results to be generalised beyond the research setting and could help inform understanding of potential uptake if MCED screening is implemented in England. There are nevertheless some limitations. The survey was set up so that the proportion of people from ethnic minority backgrounds represented the proportion in the English population. However, this meant there were relatively small numbers of participants from each ethnic minority background. Consequently, we were not able to consider variation within broader ethnic minority groups. For example, there is evidence that within South Asian minority groups, uptake of existing screening is lowest among participants from Bangladeshi backgrounds [49]. In addition, since the survey was text-based and in English, those who were unable to read English would not have been able to take part. A deeper understanding of attitudes to MCED screening in different ethnic minority communities and non-English speakers will be important.

Data collection was completed online. There is some evidence that this type of data collection is equivalent to home-based interviewer led data collection [50], but as with all research methods there are likely to be some participation biases, for example those with low digital literacy are unlikely to participate. It is possible that there are broader inequalities in the opportunity to participate. For example, our research likely under-represents the least literate in the population. The small drop-out between engaging with the invitation (which did not describe what the survey was about) and survey completion suggests that interest in the topic area itself did not bias participation (i.e. self-selection bias). However, those on the YouGov panel may be people who are more interested in research participation broadly. It is likely that alternative methodologies (e.g. community-based face-to-face surveys, using multilingual interviewers) will be required to reach some groups and provide a more complete picture of attitudes to blood-based MCED tests across all sections of the society.

Whilst it was essential to provide participants with information about MCEDs within the study, it is still uncertain how MCED screening would be offered and what the performance characteristics of any test might be. If MCED screening is offered in a context that is very different from what was described to participants, intentions and attitudes may differ. As the potential for MCED screening develops, future work will be needed to ensure our understanding of acceptability accurately reflects the provision on offer. For most of our analyses we combined those who would not have a blood-based MCED tests and those who were unsure. These groups are likely quite different and further work to understand why people (i) actively do not want the test or (ii) are not sure is needed.

Since this was the first study in this context, we developed many of the items for the questionnaire rather than using validated scales. The items drew largely on our qualitative work as well as the existing literature in the screening field. The six scales we developed could be further validated and used in future work. This would allow for comparison of the literature as more evidence becomes available. However, since the questionnaire was developed in a UK context, the items likely exclude domains that will warrant exploration in different populations e.g. the relevance of insurance pay-outs in the US healthcare system. Engagement with PPI prior to development of the research questions would have strengthened the study.

## Conclusion

This is the first study to quantify intention to have MCED blood test screening in a population-based sample. The findings suggest that intentions to take up MCED screening in England are high. While motivation to have MCED screening appears to be equitable across socio-demographic groups, inequity in uptake will likely be driven by access and opportunity which will require further consideration if a programme is implemented. Further work is needed to explore attitudes to blood-based MCED tests in different countries with different healthcare contexts. Supporting individuals with clear and accessible information will be imperative prior to an offer of an MCED test and measures should be put in place to reduce concern surrounding MCED screening results.

## Supplementary information

Supplementary Tables

Supplementary material - Exploratory Factor Analysis

Checklist for Reporting Of Survey Studies (CROSS)

## Data Availability

The dataset and syntax will be made available upon reasonable request.
