# Supplementary material for: Intention to have blood-based multi-cancer early detection (MCED) screening: a cross-sectional population-based survey in England
Source: Br J Cancer. 2024 Aug 27;131(7):1202–11. doi: 10.1038/s41416-024-02822-4 (PMC11443085; doi:10.1038/s41416-024-02822-4)
Supplement: Supplementary file 1 — Supplementary Tables [file 41416_2024_2822_MOESM1_ESM.docx]

**Table S1: Percentage indicating each response to the barrier and facilitator items (weighted)**

|  | **Strongly disagree** | | **Disagree** | | **Neither disagree nor agree** | | **Agree** | | **Strongly agree** | |
| --- | --- | --- | --- | --- | --- | --- | --- | --- | --- | --- |
|  | n | % (95% CI) | n | % (95% CI) | n | % (95% CI) | n | % (95% CI) | n | % (95% CI) |
| Taking action for health | 8 | 0.8 (0.4-1.6) | 17 | 1.8 (1.1-2.8) | 105 | 11.0 (9.1-13.1) | 479 | 50.0 (46.9-53.2) | 349 | 36.4 (33.4-39.5) |
| Perceived necessity | 15 | 1.6 (0.9-2.6) | 28 | 2.9 (2.0-4.2) | 124 | 13.0 (11.0-15.3) | 435 | 45.4 (42.3-48.6) | 355 | 37.1 (34.0-40.2) |
| Reassurance | 10 | 1.0 (0.5-1.9) | 24 | 2.6 (1.7-3.8) | 166 | 17.4 (15.1-19.9) | 476 | 49.7 (46.5-52.9) | 282 | 29.4 (26.6-32.4) |
| Lack of necessity | 417 | 43.5 (40.4-46.7) | 404 | 42.2 (39.1-45.4) | 108 | 11.3 (9.4-13.5) | 20 | 2.1 (1.4-3.3) | 8 | 0.9 (0.4-1.7) |
| Familiarity of blood tests | 14 | 1.5 (0.9-2.5) | 41 | 4.3 (3.2-5.8) | 71 | 7.4 (5.9-9.3) | 420 | 43.9 (40.7-47.0) | 410 | 42.9 (39.8-46.0) |
| Used to blood tests | 23 | 2.4 (1.6-3.6) | 65 | 6.8 (5.3-8.6) | 137 | 14.3 (12.2-16.7) | 360 | 37.6 (34.6-40.7) | 373 | 39.0 (35.9-42.1) |
| Blood tests are quick | 1 | 0.1 (0.0-0.7) | 9 | 1.0 (0.5-1.8) | 58 | 6.0 (4.7-7.7) | 470 | 49.1 (45.9-52.2) | 420 | 43.9 (40.7-47.0) |
| Blood tests safety | 3 | 0.3 (0.1-0.9) | 4 | 0.4 (0.2-1.1) | 63 | 6.6 (5.2-8.4) | 454 | 47.4 (44.2-50.6) | 434 | 45.3 (42.2-48.5) |
| Need phobia | 406 | 42.4 (39.3-45.6) | 298 | 31.1 (28.2-34.1) | 134 | 14.0 (11.9-16.4) | 88 | 9.2 (7.5-11.2) | 32 | 3.4 (2.4-4.7) |
| Discomfort | 244 | 25.5 (22.8-28.4) | 324 | 33.9 (30.9-36.9) | 200 | 20.9 (18.4-23.6) | 165 | 17.2 (15.0-19.8) | 24 | 2.5 (1.7-3.7) |
| Painful | 295 | 30.8 (28.0-33.8) | 389 | 40.6 (37.5-43.7) | 195 | 20.4 (18.0-23.1) | 66 | 6.9 (5.5-8.7) | 12 | 1.3 (0.7-2.2) |
| Fear of blood | 467 | 48.8 (45.6-52.0) | 295 | 30.8 (27.9-33.8) | 125 | 13.0 (11.0-15.3) | 54 | 5.6 (4.3-7.3) | 17 | 1.8 (1.1-2.9) |
| Fear of result | 140 | 14.6 (12.5-17.0) | 146 | 15.2 (13.1-17.7) | 241 | 25.1 (22.5-28.0) | 349 | 36.5 (33.5-39.6) | 82 | 8.6 (6.9-10.5) |
| Cause worry | 154 | 16.1 (13.9-18.6) | 238 | 24.8 (22.2-27.7) | 259 | 27.0 (24.3-30.0) | 250 | 26.1 (23.4-29.0) | 57 | 6.0 (4.6-7.7) |
| Fear of treatment | 260 | 27.1 (24.4-30.1) | 252 | 26.3 (23.6-29.2) | 167 | 17.4 (15.1-20.0) | 232 | 24.2 (21.6-27.1) | 47 | 4.9 (3.7-6.5) |
| Difficulty getting appointment | 319 | 33.4 (30.4-36.4) | 330 | 34.5 (31.6-37.6) | 209 | 21.8 (19.3-24.6) | 82 | 8.6 (7.0-10.6) | 16 | 1.7 (1.0-2.8) |
| Difficulty getting to GP | 565 | 59.1 (55.9-62.2) | 312 | 32.6 (29.7-35.7) | 49 | 5.1 (3.9-6.7) | 25 | 2.6 (1.8-3.9) | 6 | 0.6 (0.3-1.4) |
| Too busy | 555 | 57.9 (54.8-61.0) | 333 | 34.8 (31.8-37.9) | 58 | 6.1 (4.7-7.8) | 9 | 0.9 (0.5-1.8) | 2 | 0.2 (0.1-0.9) |
| Need more information | 97 | 10.2 (8.4-12.2) | 225 | 23.6 (21.0-26.4) | 256 | 26.7 (24.0-29.6) | 336 | 35.1 (32.1-38.2) | 43 | 4.5 (3.4-6.1) |
| Trust in results | 333 | 34.8 (31.8-37.9) | 465 | 48.5 (45.4-51.7) | 133 | 13.9 (11.9-16.3) | 23 | 2.4 (1.6-3.6) | 3 | 0.3 (0.1-1.0) |
| Competing interests | 308 | 32.1 (29.2-35.2) | 348 | 36.3 (33.3-39.5) | 231 | 24.1 (21.5-26.9) | 53 | 5.6 (4.3-7.2) | 18 | 1.9 (1.2-3.0) |

CI: Confidence Interval. See protocol for full details of each item and wording: https://osf.io/ka3t7

**Table S2: Percentage indicating each response to the additional barrier items asked after additional information (weighted)**

|  | **Not at all** | | **A little** | | **Quite a bit** | | **A great deal** | |
| --- | --- | --- | --- | --- | --- | --- | --- | --- |
|  | n | % (95% CI) | n | % (95% CI) | n | % (95% CI) | n | % (95% CI) |
| Anxiety from signal | 333 | 34.8 (31.8-37.9) | 386 | 40.3 (37.2-43.4) | 156 | 16.3 (14.1-18.8) | 83 | 8.6 (7.0-10.6) |
| Not wanting further tests | 509 | 53.2 (50.0-56.4) | 260 | 27.1 (24.4-30.1) | 124 | 12.9 (11.0-15.2) | 64 | 6.7 (5.3-8.5) |
| Anxiety of further tests | 315 | 32.9 (30.0-36.0) | 400 | 41.8 (38.7-44.9) | 156 | 16.3 (14.1-18.7) | 87 | 9.1 (7.4-11.1) |
| Not wanting scans | 636 | 66.4 (63.4-69.4) | 198 | 20.7 (18.3-23.4) | 77 | 8.1 (6.5-10.0) | 46 | 4.8 (3.6-6.4) |
| Not wanting endoscopy | 334 | 34.9 (31.9-38.0) | 344 | 35.9 (32.9-39.0) | 173 | 18.1 (15.8-20.7) | 107 | 11.1 (9.3-13.3) |
| False positives | 668 | 69.8 (66.8-72.6) | 194 | 20.3 (17.8-23.0) | 59 | 6.2 (4.8-7.9) | 36 | 3.7 (2.7-5.1) |

CI: Confidence Interval. See protocol for full details of each item and wording: https://osf.io/ka3t7

**Table S3: Free-text response explaining why participants would probably or definitely not have a blood test for a range of cancers**

| **Theme** | **Quotes** |
| --- | --- |
| Test causing anxiety (n=12) | “… I don't want to have the worry of going for a test which may turn out negative”  “Because it may bring up stresses, anxieties and issues unnecessarily in advance of any possible eventuality.”  “I am afraid of it.”  “I don’t want to have the stress and worry of having unnecessary tests and the worry of the outcome…”  “I think it would frighten me…”  “I would be worried that it would show positive, even if I didn’t have Cancer, and have to go through all kinds of tests.”  “It frightens me”  “… I am not sure the anxiety and stress is worth the possible benefits.”  “Prefer to live life without undue health worries”  “Stress”  “Too nervous you might find I have cancer…”  “Too scared” |
| Not wanting to know about cancer (n=7) | “Depends which cancer is found but would rather not know”  “Don't want to know.”  “Fear of illness...”  “… There is a strong history of cancer in my family and it is something I don’t want to think about until I possibly have to…”  “… I dont want to know about cancer…”  “… to be honest I'd rather not know”  “Rather not know” |
| Perceived need for the test (n=7) | “Because I don't have any trace of it”  “Because it may bring up stresses, anxieties and issues unnecessarily in advance of any possible eventuality.”  “I don't want to get on the treatment treadmill without any symptoms.”  “Not prone to any disease”  “Once one has cancer, it will get you in the end, regardless of treatments…”  “Too old to make much difference”  “why look for things you dont now you have” |
| Wanting to live life (n=5) | “At my age I wouldn't want treatment to ruin what's left of my life”  “because i like to take life as it comes and I don't want to have the worry of going for a test which may turn out negative”  “....There is a strong history of cancer in my family and it is something I don’t want to think about until I possibly have to.I just want to enjoy my life until then”  “… I would rather just live my life now thanks”  “Prefer to live life without undue health worries” |
| Concerns about accuracy (n=4) | “I don't have smear tests or other tests as they are unreliable. I can't imagine this test would be any more reliable.”  “I would be worried that it would show positive, even if I didn’t have Cancer, and have to go through all kinds of tests.”  “is it trustworthy”  “Need to see if it works first !” |
| Not wanting treatment (n=4) | “At my age I wouldn't want treatment to ruin what's left of my life”  “Because I am 71 and do not have any desire to have treatments at my age to guarantee that I could end up alive, old and waiting to die in an old peoples home”  “I don't want to get on the treatment treadmill without any symptoms.”  “I know that I would not have chemotherapy …” |
| Blood test specific concerns (n=3) | “… fear of having blood taken.”  “I don't want the NHS sticking needles in me”  “...Also not good with needles” |
| Personal experiences of cancer (n=2) | “...There is a strong history of cancer in my family and it is something I don’t want to think about until I possibly have to…”  “I know that I would not have chemotherapy after watching my Dad go through it so to be honest I'd rather not know” |
| Competing interests (n=2) | “I already have health issues (vasculitis) which requires regular blood tests, visits to consultants, daily medication. To be honest, I have enough on my plate as it is. Sorry.”  “Stress” |
| Trust in NHS (n=2) | “I do not trust the NHS”  “I don't want the NHS sticking needles in me” |
| Value of screening in general (n=2) | “I don't have smear tests or other tests as they are unreliable. I can't imagine this test would be any more reliable.”  “I have a different approach to health.” |
| Other (n=5) | “I do not want any blood test out of blue”  “Because I am 71 and do not have any desire to have treatments at my age to guarantee that I could end up alive, old and waiting to die in an old peoples home”  “I have had enough of medical interventions and tests ( like lft for example) over the last three years…”  “Sorry misread question”  “Not sure” |

| **Table S4: Logistic regression analyses exploring the association between barrier and facilitator scales with intention to have MCED screening** | | | | | |
| --- | --- | --- | --- | --- | --- |
|  | **Intenders (n=989)** | **Non-intenders (n=59)** | **Adjusted OR (95% CI)^a^** | **F(df), p-value** |  |
|  | Mean (SD) | Mean (SD) |  |  |  |
| Health Motivation | 12.98 (2.27) | 7.20 (2.74) | 2.07 (1.73-2.47) | **64.37(1,957), <.001** |  |
| Benefits of blood tests | 13.15 (2.64) | 10.54 (2.93) | 0.96 (0.83-1.10) | 0.39(1,957), .534 |  |
| Disadvantages of blood tests | 4.14 (3.35) | 6.21 (4.02) | 1.01 (0.89-1.17) | 0.06(1,957), .803 |  |
| Fear of outcome | 5.23 (3.00) | 8.56 (2.82) | 1.01 (0.81-1.26) | 0.01 (1,957), .934 |  |
| Practical barriers | 1.98 (1.84) | 4.61 (2.51) | 0.77 (0.62-0.95) | **6.19(1,957), .013** |  |
| Concerns about a positive result | 4.27 (3.98) | 11.90 (4.40) | 0.80 (0.71-0.89) | **16.66 (1,957) <.001** |  |
| SD: Standard Deviation; OR: Odds Ratio; CI: Confidence interval. Adjusted for all other barriers and facilitators (scales and individual items).  Bold indicates significance at <.05.  **Table S5: Logistic regression analyses exploring the association between barrier and facilitator scales with intention to have MCED screening** | | | | | |
|  | **Intenders N (%)** | | **Adjusted OR (95% CI)** | **Chi-squared(df), p-value** |  |
| **Needing more information** |  | |  |  |  |
| Yes | 340 (89.76) | | 0.82 (0.36-1.89) | 0.22(1,957), .642 |  |
| No | 558 (96.49) | | 1.00 |  |  |
|  |  | |  |  |  |
| **Trust in results** |  | |  |  |  |
| Yes | 17 (62.29) | | 0.78 (0.13-4.54) | 0.08(1,957), .778 |  |
| No | 882 (94.72) | | 1.00 |  |  |
|  |  | |  |  |  |
| **Competing interests** |  | |  | 0.57(1,957), .449 |  |
| Yes | 56 (78.73) | | 1.49 (0.53-4.16) |  |  |
| No | 842 (95.04) | | 1.00 |  |  |

OR: Odds Ratio; CI: Confidence interval. Adjusted for all other barriers and facilitators (scales and individual items).

Bold indicates significance at <.05.

| **Table S6: Logistic regression analyses exploring the association between barrier and facilitator scales and items with socio-demographic characteristics (adjusted, weighted)** | | | | | | | |
| --- | --- | --- | --- | --- | --- | --- | --- |
|  | **Health motivation** | | | **Benefits of blood tests score** | | **Disadvantages of blood tests score** | |
|  | Mean (SE) | | Wald's F(df),p-value | Mean (SE) | Wald's F(df),p-value | Mean (SE) | Wald's F(df),p-value |
| **Age** |  | |  |  |  |  |  |
| 50-59 | 12.50 (0.20) | | F(2,933)=0.16, p=.850 | 12.71 (0.17) | F(2,933)=2.06, p=.128 | 4.83 (0.24) | F(2,933)=3.86,  P=.021 |
| 60-69 | 12.50 (0.19) | |  | 13.12 (0.17) |  | 4.44 (0.24) |  |
| 70+ | 12.62 (0.23) | |  | 13.18 (0.22) |  | 3.87 (0.30) |  |
|  |  | |  |  |  |  |  |
|  |  | |  |  |  |  |  |
| **Sex** |  | |  |  |  |  |  |
| Male | 12.43 (0.18) | | F(1,934)=1.60, p=.207 | 13.11 (0.15) | F(1,934)=1.50  P=.221 | 4.21 (0.22) | F(1,934)=2.42,  P=.120 |
| Female | 12.65 (0.18) | |  | 12.90 (0.15) |  | 4.55 (0.22) |  |
|  |  | |  |  |  |  |  |
| **Social Grade** | | |  |  |  |  |  |
| A-B (highest) | 12.78 (0.22) | | F(3,932)=1.23, p=.299 | 13.14 (0.22) | F(3,932)=0.89,  P=.445 | 4.10 (0.29) | F(3,932)=1.13,  P=.338 |
| C1 | 12.41 (0.18) | |  | 13.11 (0.18) |  | 4.54 (0.26) |  |
| C2 | 12.62 (0.25) | |  | 12.99 (0.20) |  | 4.26 (0.28) |  |
| D-E (lowest) | 12.36 (0.22) | |  | 12.77 (0.20) |  | 4.62 (0.26) |  |
|  |  | |  |  |  |  |  |
|  |  | |  |  |  |  |  |
| **Ethnicity** |  | |  |  |  |  |  |
| White | 12.71(0.09) | | F(1,932)=1.23, .268 | 13.06 (0.10) | F(1,934)=0.18,  P=.675 | 4.06 (0.12) | F(1,934)=2.95,  P=.086 |
| Other | 12.37 (0.29) | |  | 12.95 (0.23) |  | 4.70 (0.36) |  |
|  |  | |  |  |  |  |  |
| **Employment status** | |  |  |  |  |  |  |
| Working | 12.65 (0.20) | | F(1,934)=0.89, p=.345 | 12.74 (0.17) | F(1,934)=6.14,  P=.013 | 4.53 (0.25) | F(1,934)=1.21,  P=.272 |
| Not working | 12.44 (0.18) | |  | 13.27 (0.16) |  | 4.23 (0.22) |  |

SE: Standard Error

**Table S6 (continued): Logistic regression analyses exploring the association between barrier and facilitator scales and items with socio-demographics (adjusted, weighted)**

SE: Standard Error. Bold indicates significance at <.001.

|  | **Fear of outcome score** | | | | **Practical barriers score** | | | | | **Concerns about a positive result score** | | |
| --- | --- | --- | --- | --- | --- | --- | --- | --- | --- | --- | --- | --- |
|  | Mean (SE) | Wald's F(df),p-value | | Mean (SE) | | | Wald's F(df),p-value | | | Mean (SE) | Wald's F(df),p-value | |
| **Age** |  |  | |  | | |  | | |  |  | |
| 50-59 | 5.96 (0.21) | **F(2,933)=6.69,**  **P=.001** | | 2.40 (0.15) | | | F(2,933)=2.63  P=.073 | | | 5.17 (0.32) | F(2,933)=1.05,  P=.349 | |
| 60-69 | 5.34 (0.22) |  |  | 2.06 (0.13) | | |  |  |  | 4.98 (0.32) |  |  |
| 70+ | 4.86 (0.27) |  |  | 2.29 (0.17) | | |  |  |  | 4.58 (0.38) |  |  |
|  |  |  | |  | | |  | | |  |  | |
| **Sex** |  |  | |  | | |  | | |  |  | |
| Male | 5.23 (0.20) | F(1,934)=2.33,  P=.128 | | 2.37 (0.13) | | | F(1,934)=3.66,  P=.056 | | | 4.72 (0.29) | F(1,934)=1.68,  P=.195 | |
| Female | 5.54 (0.19) |  |  | 2.13 (0.13) | | |  |  |  | 5.09 (0.30) |  |  |
|  |  |  | |  | | |  | | |  |  | |
| **Social Grade** |  | |  | | |  | |  |  | | |  |
| A-B (highest) | 5.15 (0.25) | F(3,932)=2.51,  P=.057 | | 1.84 (0.15) | | | **F(3,932)=6.40,**  **p<.001** | | | 4.21 (0.36) | F(3,932)=3.31,  P=.020 | |
| C1 | 5.78 (0.21) |  |  | 2.20 (0.14) | | |  |  |  | 4.91 (0.33) |  |  |
| C2 | 5.15 (0.26) |  |  | 2.45 (0.17) | | |  |  |  | 5.05 (0.38) |  |  |
| D-E (lowest) | 5.46 (0.25) |  |  | 2.51 (0.16) | | |  |  |  | 5.46 (0.37) |  |  |
|  |  |  | |  | | |  | | |  |  | |
| **Ethnicity** |  |  | |  | | |  | | |  |  | |
| White | 5.21 (0.11) | F(1,934)=1.11,  P=.292 | | 2.06 (0.07) | | | F(1,934)=2.82,  P=.093 | | | 4.51 (0.15) | F(1,934)=2.47,  P=.117 | |
| Other | 5.56 (0.31) |  |  | 2.44 (0.22) | | |  |  |  | 5.31 (0.49) |  |  |
|  |  |  | |  | | |  | | |  |  | |
| **Employment status** |  | |  | | |  | |  |  | | |  |
| Working | 5.30 (0.22) | F(1,934)=0.54,  P=.463 | | 2.44 (0.14) | | | F(1,934)=6.34,  P=.012 | | | 4.69 (0.33) | F(1,934)=1.67,  P=.196 | |
| Not working | 5.48 (0.20) |  |  | 2.05 (0.13) | | |  |  |  | 5.13 (0.29) |  |  |

| **Table S7: Chi-squared_1_ and logistic regression analyses exploring differences in barrier and facilitators items scores based on socio-demographic characteristics (weighted, adjusted)** | | | | | | |
| --- | --- | --- | --- | --- | --- | --- |
|  | **Would not trust results** | | **More important things to worry about** | | **Need more information** | |
|  | n agree (%) | χ ^2^(df), p | n agree (%) | χ ^2^(df), p | n agree (%) | OR (95% CI) |
| **Age** |  |  |  |  |  |  |
| 50-59 | 11 (2.73) | χ^2^(2,1914)=0.01, p =.998 | 33 (7.97) | χ^2^(2,1914)=0.96, p =.620 | 181 (43.86) | 1.00 |
| 60-69 | 9 (2.79) |  | 25 (7.80) |  | 116 (35.87) | 0.78 (0.56-1.09) |
| 70+ | 6 (2.81) |  | 13 (5.94) |  | 82 (37.06) | 0.96 (0.64-1.44) |
|  |  |  |  |  |  |  |
| **Sex** |  |  |  |  |  |  |
| Male | 14 (3.13) | χ^2^(1,957)=0.43, p =.517 | 38 (7.58) | χ^2^(1,957)=0.03, p =.87 | 202 (40.83) | 1.00 |
| Female | 12 (2.43) |  | 34 (5.69) |  | 177 (38.28) | 1.15 (0.88-1.51) |
|  |  |  |  |  |  |  |
| **Social Grade** |  |  |  |  |  |  |
| A-B | 1 (0.42) | χ^2^(3,2854)=8.14, p =.045 | 19 (8.78) | χ^2^(3,2861)=2.98 p =.397 | 72 (33.70) | 1.00 |
| C1 | 10 (3.69) |  | 13 (5.49) |  | 122 (43.33) | 1.31 (0.90-1.92) |
| C2 | 4 (2.03) |  | 15 (6.97) |  | 80 (36.36) | 1.05 (0.69-1.58) |
| D-E | 10 (4.41) |  | 22 (8.94) |  | 106 (43.36) | 1.44 (0.98-2.11) |
|  |  |  |  |  |  |  |
| **Ethnicity** |  |  |  |  |  |  |
| White | 19 (2.26) | χ^2^(1,957)=7.39, p =.008 | 59 (7.38) | χ^2^(1,957)=3.30, p =.074 | 316 (37.02) | **1.00*** |
| Any other ethnic background | 7 (6.87) |  | 12 (11.83) | | 64 (60.51) | **2.59 (1.68-3.99)** |
|  |  |  |  |  |  |  |
| **Employment status** |  |  |  |  |  |  |
| Working | 9 (2.06) | χ^2^(1,957)=1.47, p =.231 | 35 (7.60) | χ^2^(1,957)=0.01, p =.929 | 190 (41.59) | 1.00 |
| Not working | 16 (3.35) |  | 36 (7.44) |  | 176 (36.68) | 0.89 (0.65-1.24) |

*Note: Chi- squared used for two items due to small cell size.* Bold indicates significance at <.001.
