# Supplementary material for: Intention to have blood-based multi-cancer early detection (MCED) screening: a cross-sectional population-based survey in England
Source: Br J Cancer. 2024 Aug 27;131(7):1202–11. doi: 10.1038/s41416-024-02822-4 (PMC11443085; doi:10.1038/s41416-024-02822-4)
Supplement: Supplementary file 2 — Supplementary material - Exploratory Factor Analysis [file 41416_2024_2822_MOESM2_ESM.docx]

**Exploratory Factor Analysis**

As set out in our analysis plan (<https://osf.io/b694n>) we ran an EFA to see if the number of barriers and facilitators could be reduced. All 13 barrier and facilitator items and the 8 items assessing attitudes to the procedure were included in a principal components analysis using varimax rotation. We consulted the scree plot, Eigenvalues and factor loadings and interpreted these taking content validity into account.

**Table 1: Factor loadings and varimax rotation 5-factor solution for 21 items (n=958)**

|  | **Factor loading** | | | | |  |
| --- | --- | --- | --- | --- | --- | --- |
|  | **1** | **2** | **3** | **4** | **5** | **Communality** |
| **Health motivation** |  |  |  |  |  |  |
| This test would make me feel I was doing something positive about my health | **.834** |  |  |  |  | .768 |
| I would want this test even if I felt healthy | **.832** |  |  |  |  | .796 |
| This test would give me reassurance | **.817** |  |  |  |  | .724 |
| I would not need a test if I did not have any symptoms | **-.562** |  |  |  |  | .471 |
|  |  |  |  |  |  |  |
| **Benefits of blood tests** |  |  |  |  |  |  |
| Blood tests are familiar to me |  | **.860** |  |  |  | .812 |
| I am used to having blood tests |  | **.838** |  |  |  | .774 |
| Blood tests are quick |  | **.767** |  |  |  | .720 |
| Blood tests are safe |  | **.693** |  |  |  | .673 |
|  |  |  |  |  |  |  |
| **Disadvantages of blood tests** |  |  |  |  |  |  |
| I am afraid of needles |  |  | **.830** |  |  | .739 |
| I find blood tests uncomfortable |  |  | **.829** |  |  | .743 |
| Blood tests are painful |  |  | **.749** |  |  | .651 |
| I am afraid of the sight of my own blood |  |  | **.747** |  |  | .589 |
|  |  |  |  |  |  |  |
| **Fear of outcome** |  |  |  |  |  |  |
| I would be frightened of what the test might find |  |  |  | **.861** |  | .788 |
| This test would make me worry about having cancer |  |  |  | **.824** |  | .754 |
| I would be afraid of having treatment if cancer was found |  |  |  | **.704** |  | .611 |
|  |  |  |  |  |  |  |
| **Practical barriers** |  |  |  |  |  |  |
| I would find it difficult to get an appointment at a time that suits me |  |  |  |  | **.748** | .637 |
| I would find it difficult to travel to an appointment at my GP surgery |  |  |  |  | **.742** | .606 |
| I would be too busy to have a blood test |  |  |  |  | **.640** | .615 |
| **Individual items** |  |  |  |  |  |  |
| I would need to know more about how the test works |  |  |  | .450 | .339 | .357 |
| I would not trust the blood test results | -.508 |  |  |  | .349 | .449 |
| I would have more important things to worry about than this test | -.489 |  |  |  | .338 | .357 |

Loadings <0.3 were suppressed.

**Table A2: Eigenvalues, percentages of variance and cumulative percentages for the 5-factor solution for 21 items**

| Factor | Name | **Eigenvalue** | **% of variance** | **Cumulative %** |
| --- | --- | --- | --- | --- |
| **1** | **Health Motivation** | 7.11 | 33.86 | 33.86 |
| 2 | **Benefits of blood tests** | 2.30 | 10.93 | 44.79 |
| 3 | **Disadvantages of blood tests** | 1.69 | 8.04 | 52.83 |
| 4 | **Fear of outcome** | 1.34 | 6.39 | 59.22 |
| 5 | **Practical barriers** | 1.20 | 5.70 | 64.92 |
